# Sulfur-Based Copolymeric Polyamidoamines as Efficient Flame-Retardants for Cotton

**DOI:** 10.3390/polym11111904

**Published:** 2019-11-19

**Authors:** Alessandro Beduini, Federico Carosio, Paolo Ferruti, Elisabetta Ranucci, Jenny Alongi

**Affiliations:** 1 Dipartimento di Chimica, Università degli Studi di Milano, via C. Golgi 19, 20133 Milano, Italy; alessandro.beduini@studenti.unimi.it (A.B.); paolo.ferruti@unimi.it (P.F.); elisabetta.ranucci@unimi.it (E.R.); 2 Dipartimento di Scienza Applicata e Tecnologia, Politecnico di Torino, Alessandria campus, viale T. Michel, 15121 Alessandria, Italy; federico.carosio@polito.it

**Keywords:** sulfur-based polyamidoamine copolymers, flame-retardants, functional coatings, cystine, cotton

## Abstract

The polyamidoamine derived from N,N′-methylenebisacrylamide (M) and glycine (G), M-G, has been shown to be an effective flame-retardant (FR) for cotton in horizontal flame spread tests (HFST), extinguishing the flame at 5% add-on. Its activity was attributed to its intrinsic intumescence. In vertical flame spread tests (VFST), M-G failed to extinguish the flame even at 30% add-on. Conversely, in VFST, the polyamidoamine derived from M and cystine (C), M-C, inhibited cotton combustion at 16% add-on, but in HFST failed to extinguish the flame below 12% add-on. Its activity was ascribed to the release of sulfur-containing volatiles acting as radical scavengers. In this work, the FR effectiveness of M–G_m_–C_n_ copolymers with different G/C ratio was compared with that of the M–G and M–C homopolymers and of M–G/M–C blends of the same compositions. In HFST, both copolymers and blends extinguished the flame. In particular, M–G_50_–C_50_ and (M–G/M–C)_50/50_ extinguished the flame, even at 7% add-on. In VFST, the copolymers with ≥50% M–C units, similar to M–C, inhibited cotton combustion at 16% add-on. At the same add-on, the M–G/M–C blends failed to extinguish the flame. It may be concluded that, in contrast to blends, copolymers combined the merits of both homopolymers in all tests.

## 1. Introduction

During the last two decades, the need for safe, flame-retardant finishing systems for cotton has become urgent in both industrial and academic research to explore novel synthetic strategies. Considerable effort has been focused upon enhancing the char-forming efficiency of flame-retardants (FRs) by designing new intumescent systems which are able to create a thermal barrier on fabric surfaces, thereby protecting the polymer bulk [1,2,3,4]. This approach has been employed for different polymer matrices such as polyamide 6 [5], poly(ethylene terephthalate) [6], and polypropylene [7] with promising results. The efficiency of these systems was found to increase with the addition of nanoparticles synthesized by different approaches [7]. In the case of cellulosic fabrics, organo-phosphorous FRs are currently the most promising technology in the market, working mainly in the condensed-phase as intumescent systems [4]. They primarily exhibit activity by favoring cellulose dehydration that leads to thermally-stable aromatic char [8,9,10]. Recent attempts have involved the use of UV-curable FRs [11,12,13,14], hybrid organic-inorganic FRs [15,16,17,18], triazine-based FRs [19,20,21], phosphoramidate derivatives [22,23,24,25,26,27], hydrated sodium metaborate [28] and combinations thereof with reduced graphene oxide [29], and polymeric FRs, in particular polysiloxane-based FRs [30,31]. Most efforts have focused upon improving the FR effectiveness and replacing toxic chemical products with counterparts that have low environmental impact and, hence, are more sustainable.

PAAs are a family of bioinspired synthetic polymers endowed with exceptional structural versatility. They were discovered in the late 1960s, and were, in subsequent years, extensively studied for biotechnological applications [32,33]. PAAs are generally prepared by the polyaddition of prim- or sec-amines to bisacrylamides through environmentally-friendly processes carried out in water, at room temperature, and without added catalysts. The synthetic processes leading to PAAs do not produce byproducts, and are therefore easily scalable. Nearly all conceivable bisacrylamides and prim- or sec-amines can be used as monomers, including those containing different functions as side substituents. In particular, the polyaddition of bisacrylamides with several natural α-amino acids was performed, leading to a new family of bio-inspired, PAA-related polymers named polyamidoamino acids (PAACs) [34,35,36,37,38]. PAAs carrying carboxylic, guanidine, and disulfide groups in their repeating units have shown potential as FRs for cotton fabrics. In detail, glycine-derived PAAs extinguished flames in horizontal flame spread tests (HFST) at add-ons as low as ~5% on a w/w basis, due to their intrinsic intumescent behavior [39], but failed to do so in vertical flame spread tests (VFST) even at add-ons up to 30%. In contrast, disulfide-containing PAAs inhibited cotton combustion in VFST at add-ons of as low as 12 %, but were less efficient in HFST than the best-performing PAAs [40]. Their remarkable efficacy in VFST was attributed to their ability to release sulfur-containing volatile compounds upon heating that quench the flame by acting as radical scavengers. These compounds rose vertically together with the flame, thus effectively carrying out their FR action. Analogously, the inferior activity in HFST of disulfide-containing PAAs was explained by the fact that the same volatile compounds rose orthogonally to the burning sample and, therefore, left it before fully exercising their FR action. Based on this premise, it was deemed interesting to study the potential as FR of PAA copolymers from N,N′-methylenebisacrylamide and glycine/cystine mixtures. The aim was to combine the merits of both homopolymers as surface-confined FRs for cotton. This aim was fulfilled; the purpose of this work is to report the obtained results.

## 2. Materials and Methods

### 2.1. Materials

Glycine (coded as G, 98%), L-cystine (C, >98.0%), N,N′-methylenebisacrylamide (M, 99%), lithium hydroxide monohydrate (LiOH^.^H_2_O, 98%), and HCl 1M (aqueous solution), were supplied by Sigma-Aldrich (Milano, Italy) and used as received. 

Cotton (COT) with an area density of 200 g m^−2^ was purchased from Fratelli Ballesio S.r.l. (Torino, Italy). 

### 2.2. Methods

The chemical structure of PAA copolymers was assessed by ^1^H nuclear magnetic resonance (NMR), collecting spectra in D_2_O at pH 9.0 at 25 °C using a Bruker Avance DPX-400 NMR spectrometer (Milano, Italy) operating at 400.13 MHz. The thermal stability of PAA homopolymers and copolymers and of PAA-treated fabrics was assessed by thermogravimetric analysis (TGA) in nitrogen and air, from 50 to 800 °C, upon 10 °C min^−1^ heating rate, employing a TGA/DSC 2 Star^®^ System instrument equipped by Mettler-Toledo (Milano, Italy). Samples (5 mg for fabrics and 2 mg for powders) were placed in open alumina crucibles, in either inert or oxidative atmospheres under 50 mL min^−1^ gas flow. The surface morphology of untreated and treated cotton fabrics was analyzed by a LEO-1450 VP scanning electron microscope (SEM) manufactured by Zeiss (Ramsey, NJ, USA) and operating at 15 mm working distance, under 5 kV beam voltage. A fabric piece (5 × 5 mm^2^) was fixed to conductive carbon adhesive tape and then gold-metallized. Residues of combustion tests were analyzed using a Field-Emission Scanning Electron Microscope (FE-SEM), Merlin model from Zeiss (Jena, Germany), operating at 6 mm working distance, under 3 kV beam voltage, and equipped with Energy-Dispersive X-ray Spectroscopy (EDX, Jena, Germany) to perform elemental analyses. A residue sample (5 × 5 mm^2^) was fixed to the carbon adhesive tape and then chrome-metallized prior to imaging. PAA copolymers and treated fabrics were analyzed by attenuated total reflectance (ATR) Fourier transform infrared spectroscopy (FT-IR). FT-IR/ATR spectra were recorded at room temperature, in 4000 ÷ 600 cm^−1^ range, with 32 scans and 4 cm^−1^ resolution using a Perkin-Elmer Frontier FT-IR/FIR spectrophotometer (Milano, Italy), equipped with a diamond crystal characterized by a penetration depth of 1.66 μm.

### 2.3. Synthesis of Homopolymeric PAAs

**Scheme 1:**
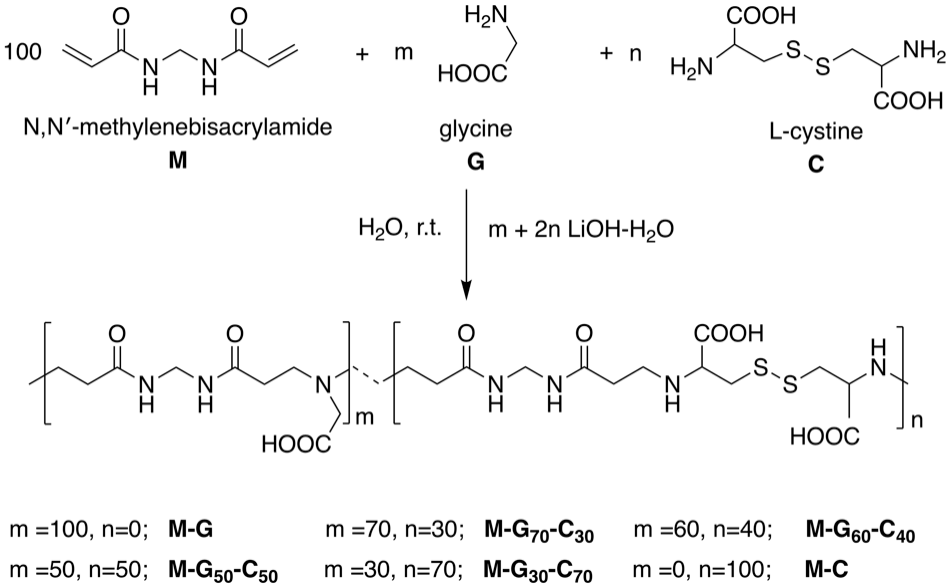
Synthesis of M–G_m_–C_n_ copolymers and the related homopolymers.

For comparison purposes, the homopolymeric PAAs named M–G and M–C were prepared in water by the reaction of N,N′-methylenebisacrylamide with glycine [39] and cystine [41,42], respectively, following previously-reported procedures. The structures of their repeat units are shown in Scheme 1; the amounts of reagents used in their synthesis are reported in Table 1.

The molecular weight of M–G was determined by Size Exclusion Chromatography (SEC). SEC traces were obtained with Toso-Haas TSK-gel G4000 PW and TSK-gel G3000 PW columns connected in series, using a Waters model 515 HPLC pump equipped with a Knauer autosampler 3800 (Knauer, Bologna, Italy), a Viscotek 270 light-scattering (LALS/RALS) detector (Malvern, Roma, Italy), and a refractive index detector (Waters, Model 2410, Milano, Italy). The mobile phase was a pH 8.00 ± 0.05 0.1 M Tris buffer solution with 0.2 M sodium chloride. The operational conditions were sample concentration 20 mg mL^−1^, flow rate 1 mL min^−1^, injection volume 20 μL, column dimensions 300 × 7.5 mm^2^, and temperature 25 °C. The instrument optical constants were determined using PEO 19 kDa as a narrow polymer standard. The sample was filtered through a 0.2 μm syringe Whatman filter before measurement. For M–G, $\bar{M_{n}}$ = 6000 was obtained, with PD = 1.4. 

### 2.4. Synthesis of Copolymeric PAAs

All copolymeric PAAs (see Scheme 1 for the structure of the repeat units) were synthesized following the same synthetic procedure and using the amounts of reagents reported in Table 1. As an example, M (3.08 g, 20 mmol) was partially dissolved in water (11.25 mL); then G (0.750 g, 10 mmol) and C (2.403 g, 10 mmol), corresponding to 0.5/0.5 G/C molar ratio, and lithium hydroxide monohydrate (1.284 g, 30 mmol) were added at room temperature (25 °C). The final mixture was gently heated at 50 °C for 15 min and then shaken until complete dissolution had occurred. The alternation of heating and shaking was completed in 2 h. Subsequently, the mixture was stirred for 8 h in the dark at 25 °C. After 48 h, the mixture was retrieved and then diluted to 50 mL with HPLC water, and the pH was adjusted to 8.5 with HCl 1M. The final product was isolated by freeze-drying. 

^1^H NMR spectra of PAA homopolymers and copolymers are reported in Figures S1–S6, Supplementary Materials. FT-IR/ATR spectra of PAA homopolymers and copolymers are reported in Figure S7.

### 2.5. Treatment of Cotton Fabrics

Cotton fabric samples of 30 × 50 mm^2^ size were impregnated twice with 7 wt.-% aqueous homopolymer or copolymer solutions at pH 9. After each deposition, samples were dried for 2 min at 90 °C. The total dry solid add-ons on cotton fabrics (*Add-on*, wt.-%) were determined by weighing each sample before (*W_i_*) and, after drying, (*W_f_*) after impregnations. The add-ons were calculated according to the Equation (1):

(1) $$dd-on=\;\frac{W_{f\;-}\;W_{i}}{W_{i}}\;\times 100$$

Strips of treated cotton will be coded COT/M–G_M–_C_n_, where m and n indicate the molar ratio between G and C. Table 2 lists the add-ons for the treated cotton fabrics under investigation.

In addition, to assess the minimum add-on for M–G_50_–C_50_ in horizontal flame spread tests, cotton stripes treated with 9, 7, and 5% add-ons were prepared, coded as COT/M–G_50_–C_50__9%, COT/M–G_50_–C_50__7% and COT/M–G_50_–C_50__5%, respectively. 

For comparison purpose, the behavior of PAA copolymers was compared with those of blends obtaining by mixing M–G and M–C solutions of the same composition, pH, and add-ons (samples COT/(M–G/M–C)_70/30_, COT/(M–G/M–C)_50/50_ and COT/(M–G/M–C)_30/70_ in Table 2). 

Strips of cotton were also treated with alternate layers of M–G and M–C by first depositing the M–G and then the M–C homopolymer (sample COT/M–G/M–C of Table 2), and then inverting the order of deposition, i.e., M–C and then M–G (sample COT/M–C/M–G of Table 2) at the same G and C content and at the same add-on. 

### 2.6. Combustion Tests 

For cotton fabrics, combustion tests in horizontal and vertical configurations were carried out by applying a 20 ± 5 mm long methane flame for 3 s to the short side of 30 × 50 mm^2^ specimens. In the horizontal configuration, the sample was positioned in a metallic frame tilted at an angle of 45° along its longer axis and then ignited. In the vertical configuration, the methane flame was applied for 3 s on the center of the short side of specimens. All specimens were conditioned to constant weight at 25 ± 1 °C, and the tests were tripled. The total combustion time (s), rate (mm s^−1^), and residual small fraction (RMF, wt.-%) were assessed.

The resistance to 35 kWm^-2^ irradiative heat flux of square fabric samples (100 × 100 mm^2^) was investigated using an oxygen-consuming cone calorimeter (Fire Testing Technology, Chichester, West Sussex, UK). Measurements were carried out in horizontal configuration, following the procedure described elsewhere [43], optimized on the basis of ISO5660 [44]. Parameters such as the time to ignition (TTI, s), peak of heat release rate (PHRR, kW m^−2^), total heat release (THR, MJ m^−2^), and residual mass fraction (RMF, wt.-%) were measured. Carbon monoxide [CO] and carbon dioxide [CO_2_] yields (both expressed in kg kg^−1^) were assessed as well. In order to establish the efficiency of the copolymer in comparison with the blend at the same add-on, the *Flame Retardancy Index* (FRI), defined as a simple yet universal dimensionless criterion born out of cone calorimetry data, was calculated. This index makes it possible to determine whether a system under investigation can be considered a “Poor”, “Good”, or “Excellent” flame retardant. FRI was calculated using the Equation (2), according to the literature [45]:

(2) $$lame\;Retardancy\;Index=\;\frac{{\left[THR\;x\;\frac{PHRR}{TTI}\right]}_{Cotton}}{{\left[THR\;x\;\frac{PHRR}{TTI}\right]}_{Treated\;otton}}$$

An FR is considered: (i) with “Poor” performance when FRI < 1, (ii) with “Good” performance when 1 < FRI < 10, and (iii) with “Excellent” performance when 10 < FRI < 100. 

Prior to the combustion tests, all specimens were conditioned to constant weight at 23 ± 1 °C for 48 h at 50% relative humidity in a climatic chamber. The experiments were performed in triplicate for each sample, calculating the experimental error.

## 3. Results and Discussion

### 3.1. Synthesis of Homopolymeric and Copolymeric PAAs

All the PAAs considered in this work were prepared following the synthetic process reported in Scheme 1. In particular, M–G and M–C were prepared by the polyaddition of N,N′-methylenebisacrylamide with glycine and L-cystine, respectively, and M–G_m_–C_n_ with glycine/L-cystine mixtures in the same proportions as those planned for the copolymers. The experimental conditions were those typically adopted for PAACs, i.e., pH 10 water solution and room temperature. The ^1^H NMR (Figures S1–S6) and FT-IR/ATR (Figure S7) spectra confirmed their structures. 

The $\bar{M_{n}}$ of M–G, as determined by SEC, was 6000 with PD = 1.4, whereas the molecular weights of the M–C and M–G_m_–M_n_ copolymers could not be determined using SEC because in the usual experimental conditions adopted for PAAs and PAACs analysis, they showed strong interactions with the stationary phase. However, the absence in the NMR spectra of all copolymers of significant peaks attributable to residual double bonds suggested that their molecular weights were at least 9000.

### 3.2. Thermal Stability of PAAs

**Figure 1:**
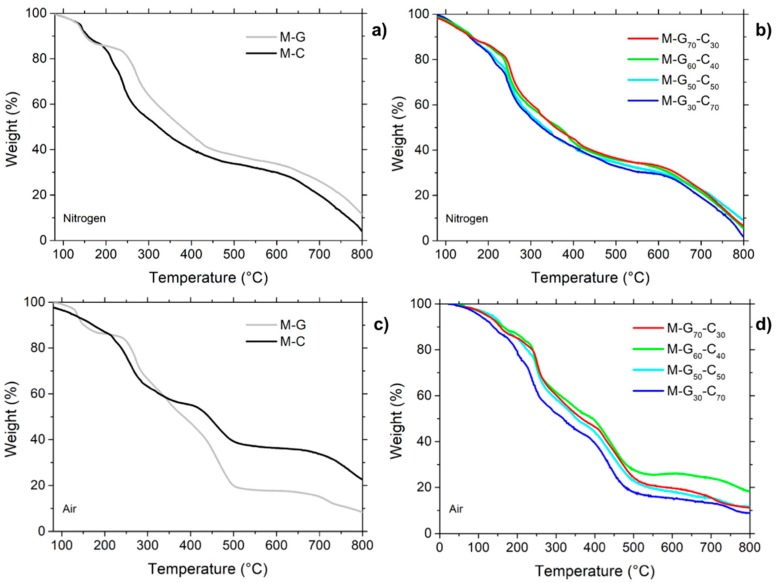
TG thermograms of PAA homopolymers and copolymers in nitrogen (**a**,**b**) and air (**c**,**d**). Add-ons: 16.0 ± 0.5%.

Figure 1 shows the TG thermograms of the M–G and M–C homopolymers, and of the M–G_m_–C_n_ copolymers, carried out in both nitrogen (a and b) and air (c and d) at between 50–800 °C. T_onset10%_, the onset decomposition temperature at 10% weight loss, T_max_, the temperature at maximum weight loss rate, and RMF, the residual mass fraction measured at 750 °C, are reported in Table 3. As previously observed with sulfur-deprived and sulfur-containing PAAs studied as FR for cotton [39,40,41], both PAA homopolymers and copolymers showed complex multimodal weight-loss curves in both nitrogen and air (Figure 1). The TG patterns in nitrogen were similar to those in air up to at least 400 °C, beyond which the oxidation of the previously-formed char occurred. More specifically, in the range of 50–200 °C, the behavior of the copolymers containing a high glycine content, namely M–G_70_–C_30_ and M–G_60_–C_40_, was similar to that of the M–G homopolymer, whereas the behavior of M–G_50_–C_50_ and M–G_30_-C_70_ was similar to that of the M–C homopolymer. 

### 3.3. FT-IR and Morphological Characterization of PAA-Treated Cotton Fabrics

Since the FRs considered in this paper are, like many PAAs, water soluble if deposited as thin films on cotton fabrics, they are almost completely washed away with water after a few washing cycles. Chemically grafting M–G–C onto a cotton surface would impart durability, and the authors are already studying a synthetic strategy to achieve this goal. This, however, should be tuned with optimized systems. Moreover, since the FR effectiveness of M–G–C copolymers and blends could be compared only if their add-ons were precisely defined, the impregnation method appeared to be the most suitable.

The fabrics were first characterized by FT-IR/ATR spectroscopy (Figure S8). The spectra of all PAA-treated cotton fabrics revealed diagnostic bands ascribed to the three components, namely 3330 (ν O-H), 2925, 2850 (ν_as_ and ν_s_ CH_2_), 1380 (δ C-H), 1320 (δ O-H), and 1018 cm^−1^ (ν C-O) for cellulose, 1610 (ν C=O) and ~1520 cm^−1^ (δ N-H), for PAA copolymers.

**Figure 2:**
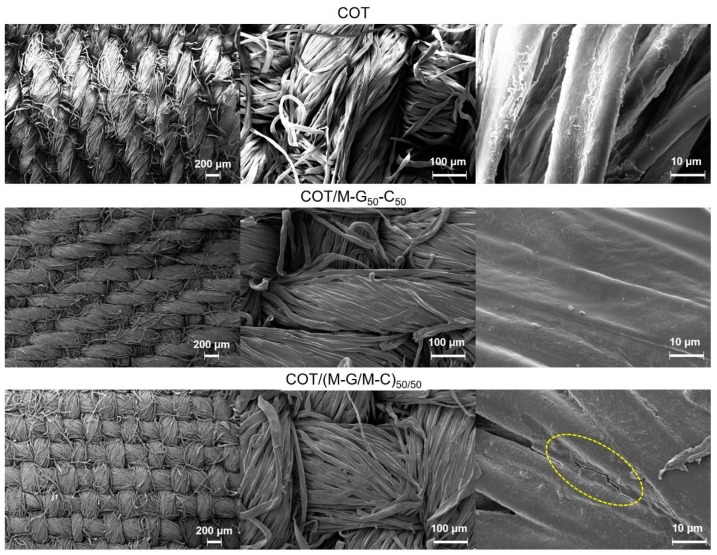
SEM micrographs of untreated-cotton fabric, and cotton fabrics treated with either M–G_50_–C_50_ copolymer (add-on: 16.0%) or (M–G/M–C)_50/50_ blend (add-on: 16.5%). The presence of cracks in the last panel is highlighted by a yellow ellipse.

Figure 2 reports the SEM micrographs of untreated cotton, cotton treated with M–G_50_–C_50_, and the corresponding blend (M–G/M–C)_50/50_. After treatment with either copolymer or blend, the fibers maintained the natural spiral nature and inhomogeneities of cotton cellulose fibrils, and the interstitial spaces among fibers were uniformly filled. Interestingly, however, while the copolymer coating was flexible and covered the fibers homogeneously, the blend formed a rigid and brittle coating, as indicated by the presence of cracks.

### 3.4. Thermal Characterization of PAA-Treated Cotton Fabrics

**Figure 3:**
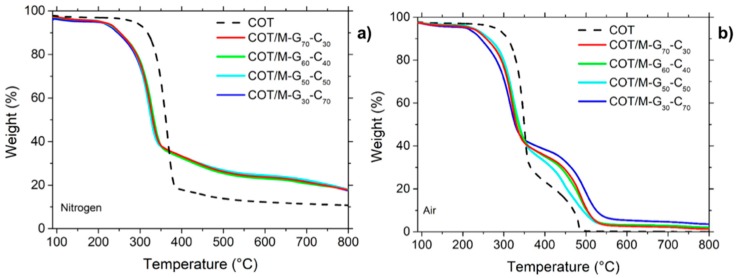
TG thermograms of PAA-treated cotton fabrics in nitrogen (**a**) and air (**b**). Add-ons: 16.0 ± 0.5%.

The thermal stability of PAA-treated cotton fabrics was investigated by TGA in nitrogen and air (Figure 3a and Figure 3b, respectively). In the 300–400 °C range, the PAA/cotton system lost more weight at lower temperatures than the untreated cotton in both nitrogen and air, as clearly evidenced by T_onset10%_ and T_max_ values reported in Table 4. However, upon decomposition, the PAA/cotton system formed a higher amount of a thermally-stable residue compared to untreated cotton (about 38–40 vs. 20–25% at T_max1_ in nitrogen and air, respectively). In nitrogen, the residue remained almost constant after further heating, while in air it oxidized. In nitrogen, the thermal decomposition of treated cotton was not significantly affected by the copolymer composition (Figure 3a), whereas in air, PAA-cotton systems with higher cystine contents anticipated decomposition to a higher extent and, upon oxidation, formed higher amounts of char.

### 3.5. Combustion Characterization of PAA-Treated Cotton Fabrics

Since, in addition to reducing the emission of sulfur oxides (see later), this work aimed to combine the merits of the M–G and M–C homopolymers, which, in previous works, were found to behave differently in horizontal and vertical flame spread tests, the effectiveness of the M–G–C copolymers was determined in both fire scenarios in order to demonstrate that they were indeed effective in both situations. Moreover, the flame retardancy of PAA-treated cotton fabrics was also studied in terms of resistance to an irradiative heat flux (35 kwm^−2^) by oxygen consumption cone calorimetry.

#### 3.5.1. Vertical Flame Spread Tests

**Figure 4:**
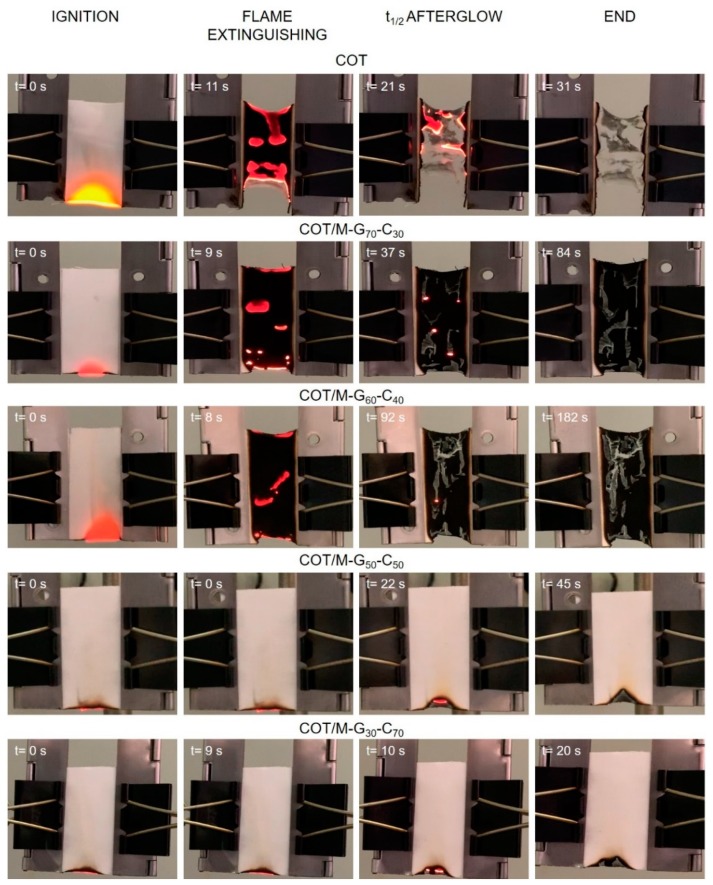
Snapshots of untreated, copolymeric PAA-treated cotton in vertical flame spread tests. Add-ons: 16.4, 16.3, 16.0 and 16.2% for COT/M–G_70_–C_30_, COT/M–G_60_–C_40_, COT/M–G_50_–C_50_ and COT/M–G_30_–C_70_, respectively.

When a flame was applied to untreated cotton in vertical configuration, it vigorously and completely burned, without leaving any residue at the end of the test (Figure 4 and Table 5).

When cotton was treated with copolymers that were rich in glycine, such as in COT/M–G_70_–C_30_ and COT/M–G_60_–C_40_, the flame glided on the surface of the fabrics but did not self-extinguish. Moreover, the combustion time was increased by afterglow. Conversely, copolymers with high cystine content, namely M–G_50_–C_50_ and M–G_30_–C_70_, behaved similarly to M–C, and in their presence, cotton fabrics did not ignite. Only afterglow was observed, which disappeared after about 20 s. As previously postulated for M–C, [39,40], even copolymers with high cystine contents acted as efficient FR since, upon thermal decomposition, they released in the gas-phase sulfur volatiles that quenched the radicals released by cotton decomposition in the gas phase and, therefore, that hindered flame propagation.

**Figure 5:**
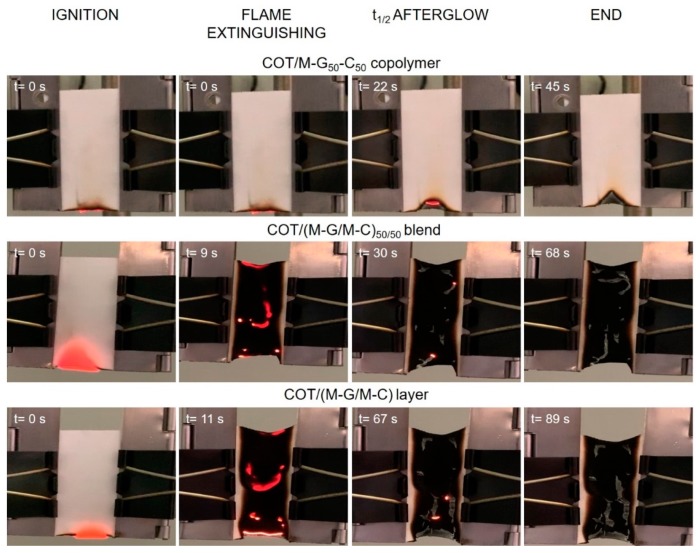
Snapshots of untreated, cotton fabrics trated with M–G_50_–C_50_ copolymer (add-on: 16.0%), (M–G/M-C)_50/50_ blend (add-on: 16.5%), and M–G/M–C layer (add-on: 16.1%) in vertical flame spread tests.

The performance of cotton treated with the M–G_m_–C_n_ copolymers was compared with that of fabrics treated with M–G/M–C blends of different compositions. The FR performance of the M–G_50_–C_50_ coating, which, together with M–G_30_-C_70_, proved in VFST to be the most effective, was compared with that of the (M–G/M–C)_50/50_ blend and those of the layered coatings obtained by alternatively depositing M–G and M–C layers (samples COT/M–G/M–C and COT/M–C/M–G in Table 5). It turned out that in VFST, neither the M–G/M–C blends, including those with 30/70 G/C content, nor the layered M–G/M–C coatings were capable of extinguishing the flame, as shown in Figure 5 for the case of the 50:50 G/C composition and in Figure S9 for all other cases. These results demonstrate that in VFST tests, the cystine and glycine units must be intimately connected by a common polymer chain to exert a cooperative FR action, whereas the spatial proximity achieved by drying mixed solutions of the two homopolymers was not sufficient. A possible explanation is that if M–C and M–G form different polymer chains, each chain reacts independently to flame application. If one of the two, probably the M–G chain, is prone to start burning, it acts as starter for the combustion of the other. This cannot easily take place when the two units are distributed along a single polymer chain.

#### 3.5.2. Horizontal Flame Spread Tests (HFST)

**Figure 6:**
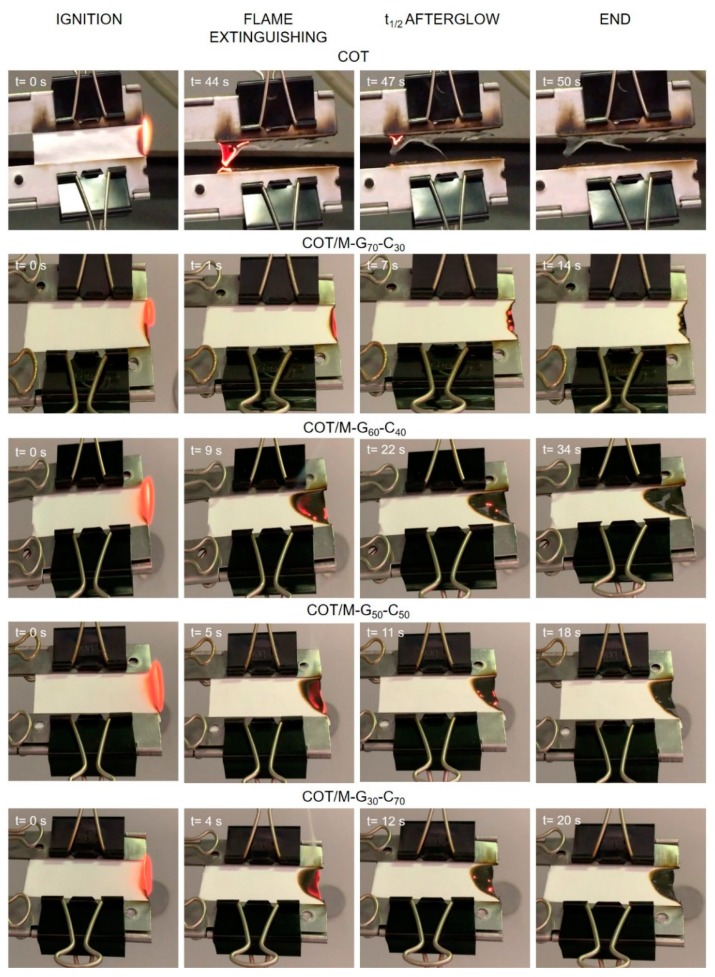
Snapshots of untreated, copolymeric PAA-treated cotton in horizontal flame spread tests. Add-ons: 16.4, 16.3, 16.0, and 16.2% for COT/M–G_70_–C_30_, COT/M–G_60_–C_40_, COT/M–G_50_–C_50_ and COT/M–G_30_–C_70_, respectively.

In HFST, untreated cotton quickly and completely burned, without leaving any residue (Figure 6 and Table 6). Conversely, all COT/M–G_n_–C_m_ samples self-extinguished at 16% add-on, regardless of composition, in the composition range studied (glycine content ranging in between 30–70% on a molar basis). No remarkable differences were observed in terms of the combustion time (Table 6), and only modest differences were observed in terms of RMF. COT/M–G_70_–C_30_, containing the highest glycine content, left the highest residue (see also Figure 6). These findings are in line with the previous data [39,40,41] showing that, in HFST, sulfur-deprived PAAs are more efficient than SS-PAAs.

**Figure 7:**
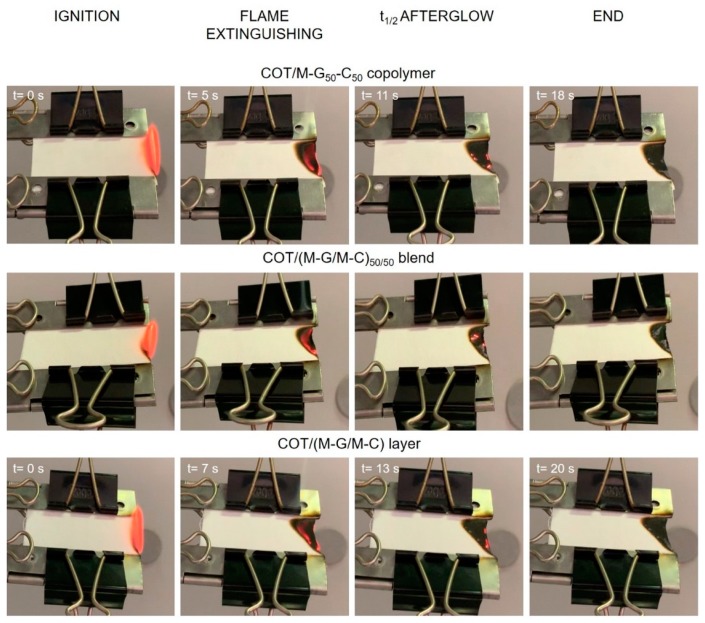
Snapshots of untreated, cotton fabrics treated with M–G_50_–C_50_ copolymer (add-on: 16.0%), (M–G/M–C)_50/50_ blend (add-on: 16.5%), and M–G/M–C layer (add-on: 16.1%) in horizontal flame spread tests.

The behavior of COT/M–G_50_–C_50_ was also compared with that of COT/(M–G/M–C)_50/50_ and COT/M–G/M–C in HFST; no significant differences were observed (Figure 7 and Table 6).

**Figure 8:**
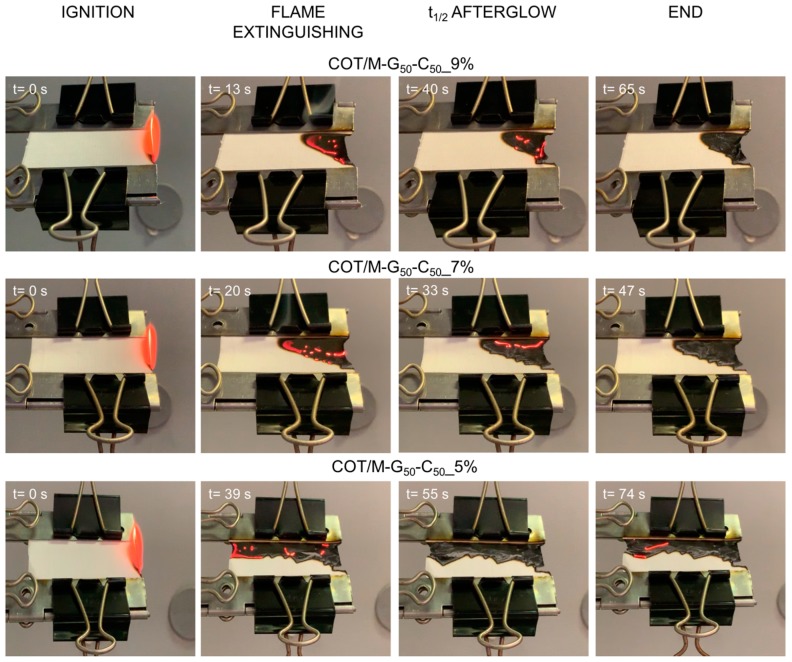
Snapshots of cotton fabrics treated with 9%, 7% and 5% add-ons of M–G_50_–C_50_ copolymer in horizontal flame spread tests.

In HFST, the minimum add-on required for inducing self-extinguishment was assessed for COT/M–G_50_–C_50_. The results, shown in Figure 8, demonstrated that at add-on 7%, COT/M–G_50_–C_50_ ignited, but self-extinguished with a 40 s afterglow, whereas 5% add-on was not sufficient to completely block the cotton combustion. The specimens burned, but only partially. At 9% add-on, M–G_50_–C_50_ stopped cotton combustion, leaving a lower RMF. These results should be compared with the 16% minimum add-on observed for M–C [41] and 5% for M–G homopolymers [39]. Therefore, the HFST performance of COT/M–G_50_–C_50_ was intermediate, but only slightly inferior to that of the M–G homopolymer.

#### 3.5.3. Cone Calorimetry Tests

**Figure 9:**
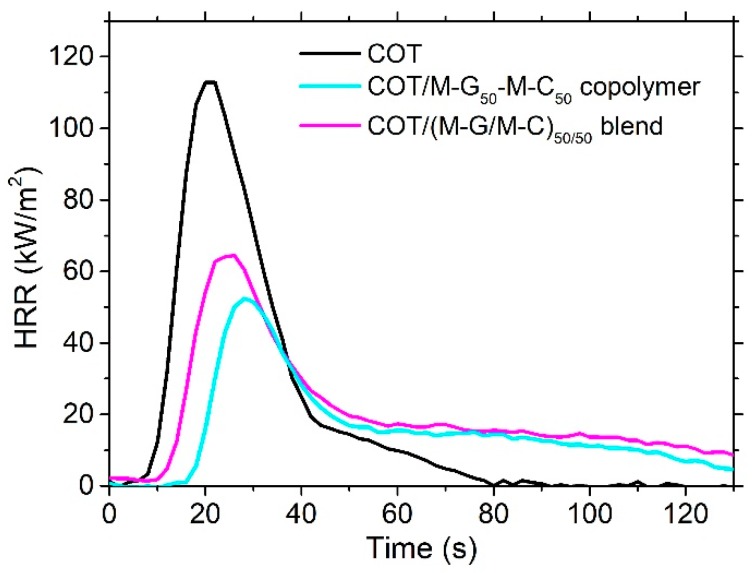
HRR curves of untreated, cotton fabrics treated with the M–G_50_–C_50_ copolymer (add-on: 16.0%) and (M–G/M–C)_50/50_ blend (add-on: 16.5%) by cone calorimetry tests.

Since the M–G_50_–C_50_ copolymer turned out to be the most efficient FR for cotton fabrics in VFST, the resistance to an irradiative heat flux of 35 kw m^−2^ by cone calorimetry was determined on cotton treated with this sample and its performance compared with that of cotton treated with an (M–G/M–C)_50/50_ blend, and untreated cotton. Figure 9 reports the corresponding HRR curves, and Table 7 lists the collected data.

In addition to a small TTI increase, both copolymer and blend drastically decreased the PHRR of cotton (Figure 9) and increased the final residue (RMF in Table 7). However, the M–G_50_–C_50_ copolymer worked better than the (M–G/M–C)_50/50_ blend, reducing PHRR by 55% vs. 44%, respectively, and exhibiting a higher FRI value (Table 7). In fact, the FRI of cotton treated with the copolymer was more than doubled compared to that of cotton treated with the corresponding blend (3.6 vs. 1.7). Furthermore, the copolymer released the lowest average amounts of CO and CO_2_ (Table 7), with a reduction of 20 and 29% compared to untreated cotton.

#### 3.5.4. Morphological Characterization and EDX Elemental Analysis of Combustion Residues

**Figure 10:**
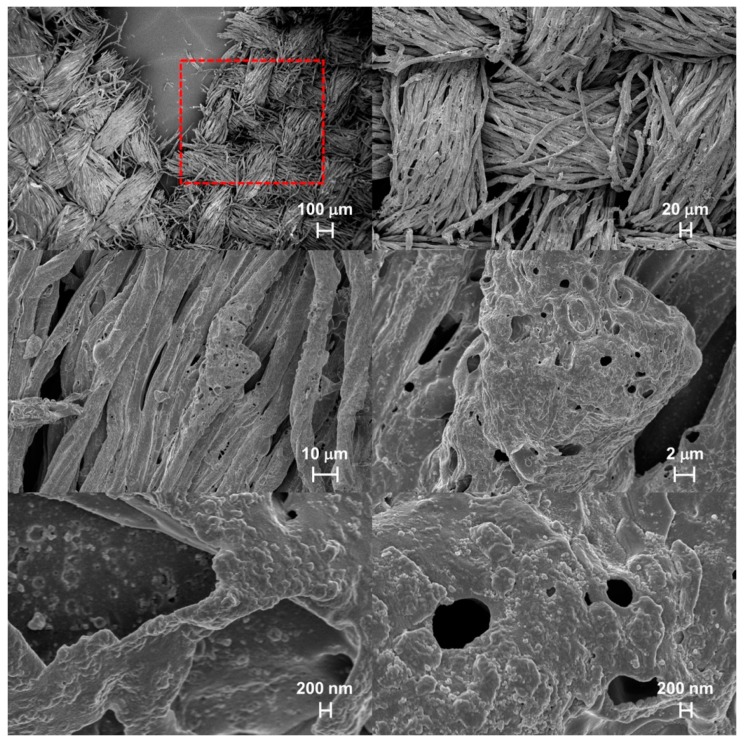
FE-SEM micrographs of COT/M–G_50_–C_50_ residue after vertical flame spread tests.

**Figure 11:**
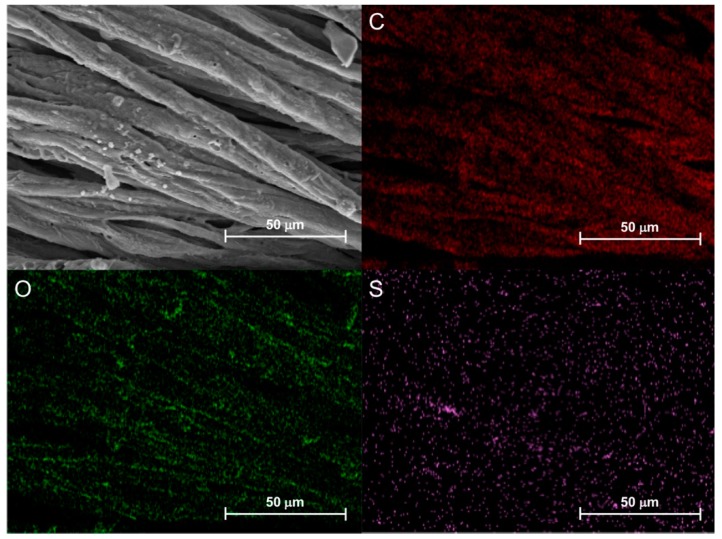
EDX analysis of COT/M–G_50_–C_50_ residue after vertical flame spread tests. Distribution of carbon (C), oxygen (O), and sulfur (S).

**Figure 12:**
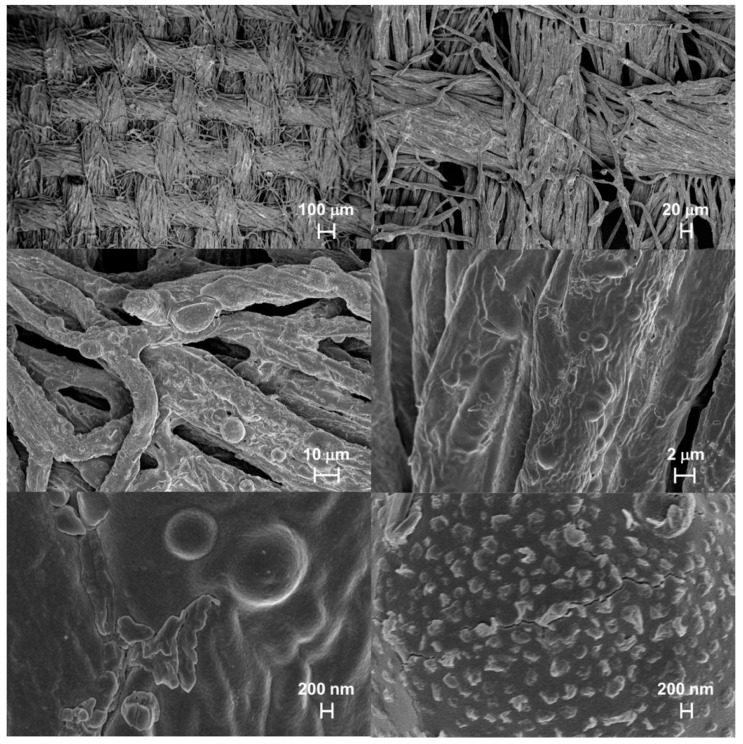
FE-SEM micrographs of COT/M–G_50_–C_50_ residue after horizontal flame spread tests.

**Figure 13:**
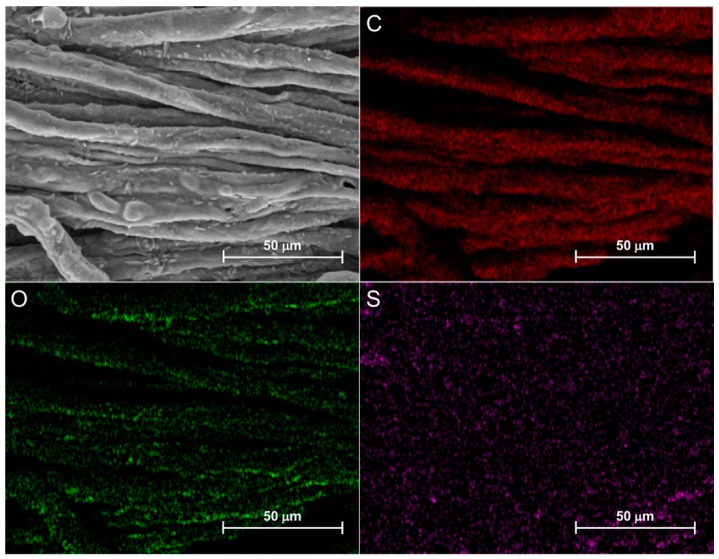
EDX analysis of COT/M–G_50_–C_50_ residue after horizontal flame spread tests. Distribution of carbon (C), oxygen (O), and sulfur (S).

The M–G_50_–C_50_ copolymer turned out to be the most efficient FR system for cotton fabrics in vertical flame spread and cone calorimetry tests. In order to deeply investigate the mechanisms through which this copolymer works, a morphological characterization of the residues after combustion tests was carried out was performed using FE-SEM and EDX elemental analysis. Figure 10 reports several magnifications of the small area (highlighted by a red square) consumed during VFST (Figure 4). In this scenario, the specimen did not ignite, but underwent pyrolysis, and only a very small area of the sample was consumed by afterglow for 24 s, leaving a RMF of 95% (Table 5). In Figure 10, it is possible to distinguish the original texture of the cotton fabrics, as well as the presence of a coating dense and rich in intumescent bubbles, demonstrating the intumescent features of this copolymer, according to that previously observed for homopolymeric PAAs [39,40]. An EDX analysis of the COT/M–G_50_–C_50_ residue after the VFST shown in Figure 11 showed homogeneous distribution and fine dispersion of elemental sulfur in the whole investigated area, in addition to carbon and oxygen. This suggested that the cystine groups present in the repetitive unit of the copolymer were active not only in the gas-phase, as already discussed above, but also in the condensed-phase. An identical morphology of the COT/M–G_50_–C_50_ residue and sulfur distribution and dispersion in the investigated area were observed after HFST, and are visible in Figure 12 and Figure 13, respectively. In this case, the sample ignited (Figure 6) but burned for only 27 s (Table 6), reaching self-extinguishment and leaving a residue of 85%. Also in this fire scenario, and less drastic with respect to the description of the vertical configuration, the M–G_50_–C_50_ copolymer turned out to be active in the gas-phase, quenching the radicals sustaining the combustion cycle, but, at the same time, they were efficient in the condensed-phase, generating an intumescent char structure that helped suppress cotton combustion. Elemental sulfur was also found in this residue (Figure 13), suggesting an active role of cystine groups to form this structure, analogously to what observed in VFST.

**Figure 14:**
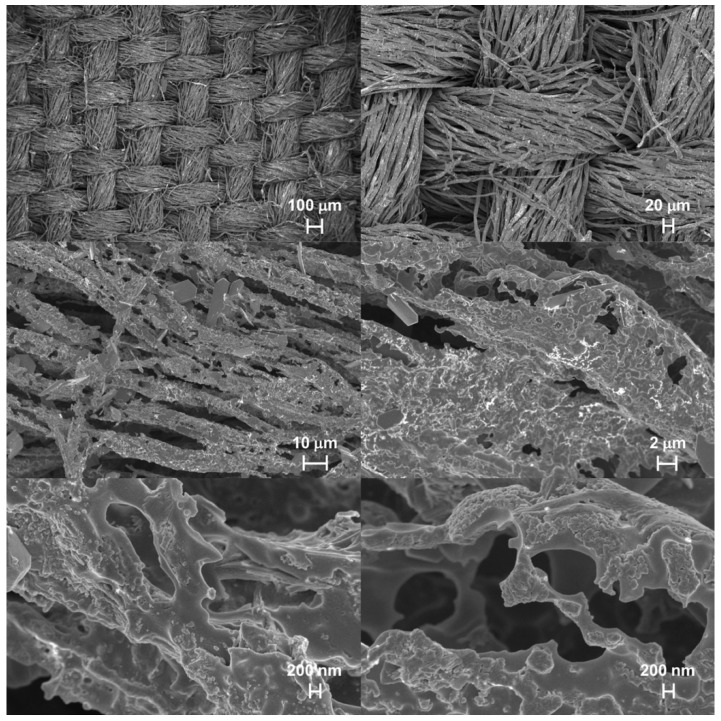
FE-SEM micrographs of COT/M–G_50_–C_50_ residue after cone calorimetry tests.

**Figure 15:**
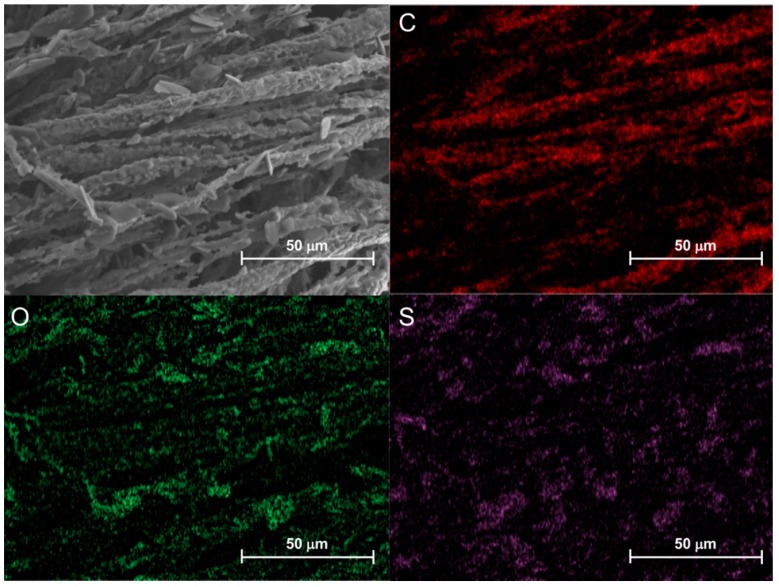
EDX analysis of COT/M–G_50_–C_50_ residue after cone calorimetry tests. Distribution of carbon (C), oxygen (O), and sulfur (S).

In the case of cone calorimetry, the morphology observed (Figure 14) was slightly different than those previously observed in flame spread tests, since the sample ignited under 35 kwm^-2^ heat flux and burnt vigorously, leaving a RMF of 8%. Also in this case, the texture of the pristine cotton fabrics was observed, as well as the presence of a consistent coating that completely covered the burnt fibers; however, the presence of intumescent bubbles was less pronounced. This can be ascribed to the explosion of bubbles due to the well-ventilated conditions typical of cone calorimetry tests. However, even if the effect in the gas-phase of cystine groups was almost negligible, as demonstrated by the small increase of TTI registered (18 vs. 12 s for COT/M–G_50_–C_50_ and COT, respectively, Table 7), the presence, distribution, and dispersion of sulfur in the combustion residue, demonstrated by EDX analysis (Figure 15), confirmed the hypothesis of activity also in the condensed-phase. This also demonstrated that the sulfur dioxide released during combustion was significantly lower than the theoretical one, as calculated for 100% conversion. On the other hand, the burned portion of the treated cotton released, in theory, SO_2_ in a quantity equal to 0.21% and 0.675% of the sample weight. If, under extreme conditions, the whole specimen burned, it would release 5.2% SO_2_. Since also the M–G_50_–C_50_ copolymer did not ignite at 16% add-on, leaving 95% RMF in both VFST and HFST tests, in theory, they could release SO_2_ in a quantity equal to 0.14% of the sample weight. Again, if, under extreme conditions, the whole specimen burned, it could release up to 2.83% SO_2_. This demonstrates that under the operational conditions, the release of SO_2_ would have been modest.

## 4. Conclusions

Following the results of previous research on the FR activity of PAAs of different structure, and, particularly, the outstanding performance of M–G and M–C homopolymers in HFST and VFST, respectively, a small library of M–G_m_–C_n_ copolymers of various compositions was prepared and tested in both HFST and VFST, as well as in cone calorimetry tests. The results were compared with those obtained from (M–G/M–C) blends of the same compositions in the same tests.

In VFST tests, the M–C homopolymer inhibited cotton ignition at 16% add-on, whereas M–G failed to extinguish the flame even at 30% add-on. In this work, it was found that the copolymers with C contents greater than 50% performed essentially as well as the corresponding M–C homopolymer. Conversely, all corresponding blends failed to extinguish the flame at the same minimal add-on at which the M–G_m_–C_n_ copolymers and M–C homopolymer did. These results are in line with the data obtained from cone calorimetry tests, where, for instance, the PHRR was reduced by 55% vs. 44%, passing from cotton to COT/M–G_50_–C_50_ and COT/(M–G/M–C)_50/50_, respectively.

Unlike the VSFT test, in the HFST test, the M–G homopolymer showed significantly better performance than M–C. In particular, they extinguished the flame at an add-on of 5% and 12% respectively. In this work, it was found that the M–G_50_–C_50_ copolymer and the 50/50 blend (M–G /M–C) extinguished the flame at an add-on of 7%.

The morphological characterization and elemental analysis of the COT/M–G_50_–C_50_ residues after combustion tests, performed by FE-SEM and EDX analysis, showed the substantial presence of finely-dispersed and homogeneously-distributed sulfur, suggesting its role not only in the gas-phase, but also in the condensed-phase of combustion. This demonstrated that the sulfur dioxide released during combustion was significantly lower than the theoretical maximum, i.e., as calculated for 100% conversion.

From the above results, it may be reasonably concluded that the copolyaddition of G and C with M is an excellent approach by which to obtain particularly effective polymeric FRs combining the merits of both homopolymers.

## Figures and Tables

**Table 1:** Chemical composition and amounts of reagents used in PAA synthesis.

PAA	G/C Molar Ratio	[M] (g, mmol)	[G] (g, mmol)	[C] (g, mmol)	LiOH·H_2_O (g, mmol)	H_2_O (mL)	N (%)	S (%)
M–G	-	3.08, 19.98	1.49, 19.98	-	0.87, 20.07	8.10	18.3	-
M–C	-	1.54, 9.99	-	2.41, 9.99	0.86, 20.50	7.00	14.2	16.2
M–G_70_–C_30_	0.7/0.3	1.04, 6.75	0.35, 4.72	0.49, 2.05	0.37, 8.82	3.30	16.6	6.9
M–G_60_–C_40_	0.4/0.6	1.54, 9.99	0.45, 5.99	0.96, 3.99	0.60, 14.30	5.30	16.1	8.7
M–G_50_–C_50_	0.5/0.5	1.01, 6.55	0.24, 3.20	0.77, 3.22	0.42, 10.00	3.67	15.7	10.3
M–G_30_–C_70_	0.3/0.7	1.01, 6.55	0.15, 1.99	1.09, 4.54	0.48, 11.44	4.15	15.0	13.0

**Table 2:** Add-ons assessed for PAA-treated cotton fabrics.

Cotton Sample	Add-on ^1^ (%)
**Copolymers**	
COT/M–G_70_–C_30_	16.4
COT/M–G_60_–C_40_	16.3
COT/M–G_50_–C_50_	16.0
COT/M–G_30_–C_70_	16.2
**Blends**	
COT/(M–G/M–C)_70/30_	16.2
COT/(M–G/M–C)_50/50_	16.5
COT/(M–G/M–C)_30/70_	16.5
**Layers**	
COT/M–G/M–C	16.1
COT/M–C/M–G	16.2

^1^ Add-on ±0.5%.

**Table 3:** Thermal data of homopolymeric and copolymeric PAAs in nitrogen and air by thermogravimetric analysis.

PAA	Add-on (%)	T_onset10%_^1^ (°C)	T_max1_^2^ (°C)	T_max2_^3^ (°C)	RMF at 750 °C ^4^ (%)
Nitrogen					
M–G	16.0	146	144, 268 ^5^	-	11.5
M–C	16.0	154	146, 208, 246 ^5^	-	4.0
M–G_70_–C_30_	16.4	152	248^5^, 322, 410	-	6.5
M–G_60_–C_40_	16.3	159	246^5^, 386, 410	-	5.5
M–G_50_–C_50_	16.0	158	213, 253^5^, 408	-	9.5
M–G_30_–C_70_	16.2	149	213, 243^5^, 408	-	2.0
Air					
M–G	16.0	148	139, 272 ^5^	468	8.5
M–C	16.0	170	221, 225, 250 ^5^	449	22.0
M–G_70_–C_30_	16.4	154	155, 251 ^5^	445	11.5
M–G_60_–C_40_	16.3	161	156, 243	444	18.0
M–G_50_–C_50_	16.0	161	162, 253 ^5^	449	12.0
M–G_30_–C_70_	16.2	138	201, 240 ^5^	449	9.0

^1^ Onset decomposition temperature at 10% weight loss. ^2^ First temperature at maximum weight loss rate from dTG curves. ^3^ Second temperature at maximum weight loss rate from dTG curves. ^4^ Residual mass fraction at 750 °C. ^5^ Main decomposition event.

**Table 4:** Thermal data of PAA-treated cotton fabrics in nitrogen and air by thermogravimetric analysis.

Sample	Add-on (%)	T_onset10%_^1^ (°C)	T_max1_^2^ (°C)	T_max2_^3^ (°C)	RMF at 750 °C ^4^ (%)
	**Nitrogen**				
COT	-	319	365	-	12.0
COT/M–G_70_–C_30_	16.4	251	327	-	18.0
COT/M–G_60_–C_40_	16.3	251	329	-	18.0
COT/M–G_50_–C_50_	16.0	251	328	-	18.0
COT/M–G_30_–C_70_	16.2	250	329	-	18.0
	**Air**				
COT	-	304	345	480	0
COT/M–G_70_–C_30_	16.4	256	319	487	1.5
COT/M–G_60_–C_40_	16.3	256	319	492	2.0
COT/M–G_50_–C_50_	16.0	264	322	444	2.0
COT/M–G_30_–C_70_	16.2	242	317	500	4.0

^1^ Onset decomposition temperature at 10% weight loss. ^2^ First temperature at maximum weight loss rate from dTG curves. ^3^ Second temperature at maximum weight loss rate from dTG curves. ^4^ Residual mass fraction at 750 °C.

**Table 5:** Combustion data of untreated and PAA-treated cotton fabrics from vertical flame spread tests.

Sample	Add-on (%)	Note	Combustion Time ^1^ (s)	Extinguishment (YES/NO)	RMF ^2^ (%)
COT	-	Flaming	33	NO	2
**Cotton fabrics treated with PAA copolymers**					
COT/M–G_70_–C_30_	16.4	Flaming	76	NO	31
COT/M–G_60_–C_40_	16.3	Flaming	191	NO	41
COT/M–G_50_–C_50_	16.0	No flaming, only afterglow	24	YES	95
COT/M–G_30_–C_70_	16.2	No flaming, only afterglow	23	YES	96
**Cotton fabrics treated with PAA blends**					
COT/(M–G/M–C)_70/30_	16.2	Flaming	74	NO	27
COT/(M–G/M–C)_50/50_	16.5	Flaming	58	NO	32
COT/(M–G/M–C)_30/70_	16.5	Flaming	82	NO	35
**Cotton fabrics treated with PAA layers**					
COT/M–G/M–C	16.1	Flaming	73	NO	37
COT/M–C/M–G	16.2	Flaming	112	NO	29

^1^ Combustion time ±1 s. ^2^ RMF ±1.0%.

**Table 6:** Combustion data of untreated and PAA-treated cotton fabrics from horizontal flame spread tests.

Sample	Add-on (%)	Combustion Time ^1^ (s)	Extinguishment (YES/NO)	RMF ^2^ (%)
COT	-	50	NO	1
	**Cotton fabrics treated with PAA copolymers**			
COT/M–G_70_–C_30_	16.4	28	YES	94
COT/M–G_60_–C_40_	16.3	27	YES	82
COT/M–G_50_–C_50_	16.0	27	YES	85
COT/M–G_30_–C_70_	16.2	21	YES	88
	**Cotton fabric treated with PAA blend**			
COT/(M–G/M–C)_50/50_	16.5	34	YES	85
	**Cotton fabric treated with PAA layer**			
COT/M–G/M–C	16.2	23	YES	80

^1^ Combustion time ± 1 s. ^2^ RMF ± 1.0%.

**Table 7:** Combustion data of untreated and cotton fabrics treated with M–G_50_–C_50_ copolymer and (M–G/M–C)_50/50_ blend by cone calorimetry tests.

Sample	Add-on (%)	TTI ^1^ (s)	PHRR ^2^ (kw m^−2^)	FRI ^3^	THR ^4^ (MJ m^−2^)	[CO] (kg kg^−1^)	[CO_2_] (kg kg^−1^)	RMF ^5^ (%)
COT	-	12 ±4	116 ±6	-	2.2 ±0.1	0.065 ±0.003	1.90 ±0.01	-
COT/M–G_50_–C_50_ copolymer	16.0	18 ±1	53 ± 3 (−55%) ^6^	3.6	2.0 ±0.1	0.052 ±0.003 (−20%) ^6^	1.35 ±0.01 (−29%) ^6^	8.0 ± 0.5
COT/(M–G/M–C)_50/50_ blend	16.5	14 ±1	66 ± 3 (−44%) ^6^	1.7	2.6 ±0.1	0.063 ±0.003 (−4%) ^6^	1.61 ±0.01 (−15%) ^6^	7.0 ± 0.5

^1^ Time to ignition. ^2^ Heat release rate peak. ^3^ Flame Retardancy Index. ^4^ Total heat release. ^5^ Residual mass fraction. ^6^ Reduction % with respect to untreated cotton.

## Supplementary Materials

The following are available online at https://www.mdpi.com/2073-4360/11/11/1904/s1. Figure S1: ^1^H NMR spectrum of M-G homopolymer, Figure S2: ^1^H NMR spectrum of M-C homopolymer, Figure S3: ^1^H NMR spectrum of M-G_70_-C_30_ copolymer, Figure S4: ^1^H NMR spectrum of M-G_60_-C_40_ copolymer, Figure S5: ^1^H NMR spectrum of M-G_50_-C_50_ copolymer, Figure S6: ^1^H NMR spectrum of M-G_30_-C_70_ copolymer, Figure S7: FT-IR/ATR of PAA homopolymers and copolymers, Figure S8: FT-IR/ATR of cotton untreated and treated with copolymeric PAAs, Figure S9: Snapshots of cotton fabrics treated with (M-G/M-C)_70/30_, (M-G/M-C)_50/50_ and (M-G/M-C)_30/70_ blends in vertical flame spread tests.
