# Supplementary material for: Sulfur-Based Copolymeric Polyamidoamines as Efficient Flame-Retardants for Cotton
Source: Polymers (Basel). 2019 Nov 19;11(11):1904. doi: 10.3390/polym11111904 (PMC6918177; doi:10.3390/polym11111904)
Supplement: Supplementary file 1 [file polymers-11-01904-s001.pdf]

Supplementary Materials

Article

Sulfur-based Copolymeric Polyamidoamines as Efficient Flame-Retardants for Cotton

Alessandro Beduini [1], Federico Carosio [2], Paolo Ferruti [1], Elisabetta Ranucci [1] and Jenny Alongi [1,] *

[1] Dipartimento di Chimica, Università degli Studi di Milano, via C. Golgi 19, 20133 Milano, Italy; alessandro.beduini@studenti.unimi.it (A.B.), paolo.ferruti@unimi.it (P.F.), elisabetta.ranucci@unimi.it (E.R.),

[2] Dipartimento di Scienza Applicata e Tecnologia, Politecnico di Torino, Alessandria campus, viale T. Michel 15121 Alessandria, Italy; federico.carosio@polito.it

* Correspondence: jenny.alongi@unimi.it; Tel.: +39-0250314108

Figures S1-S6: ^{1}H NMR spectra of PAA homopolymers and copolymers.

Figures S7 and **S8**: FT-IR/ATR spectra of PAAs and PAA-treated cotton fabrics.

Figure S9: Snapshots of PAA blends from VFST.

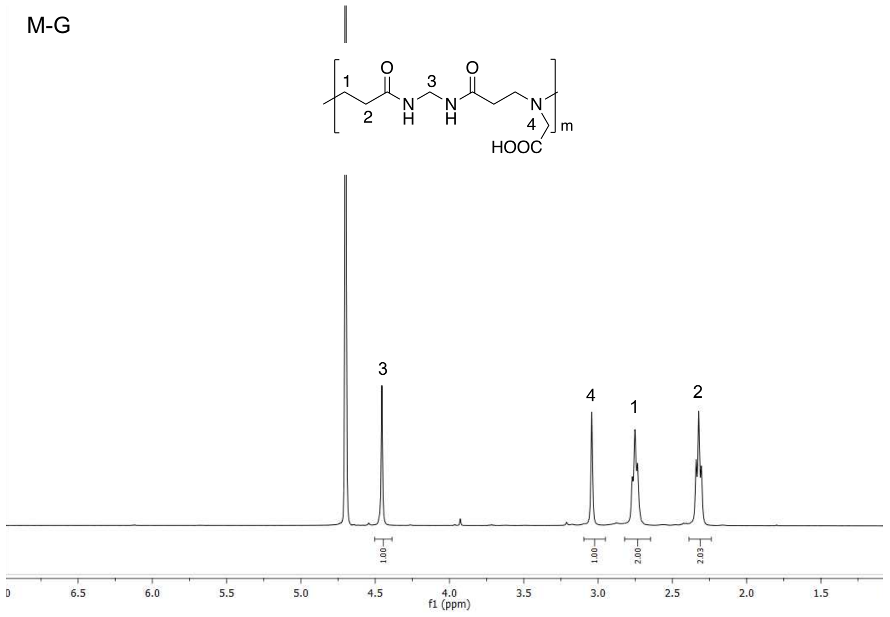

Figure S1. ^{1}H NMR spectrum of M-G homopolymer.

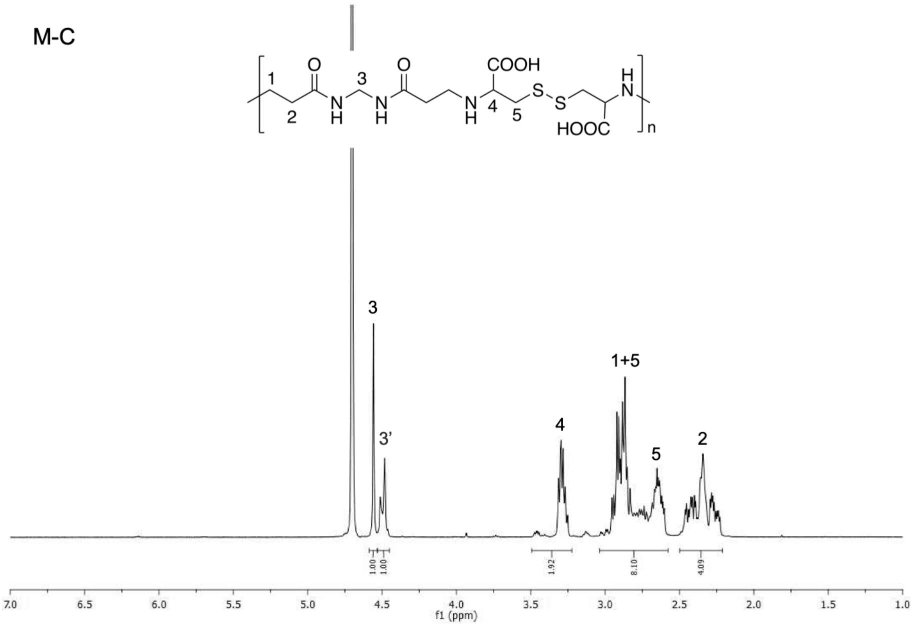

Figure S2. ^{1}H NMR spectrum of M-C homopolymer.

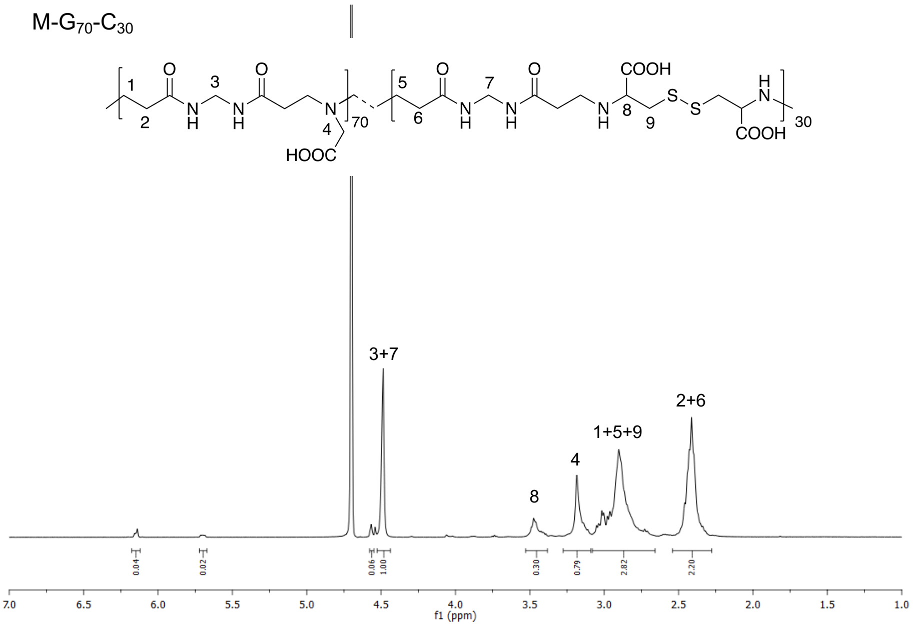

Figure S3. ¹H NMR spectrum of M-G_{70}-C_{30} copolymer.

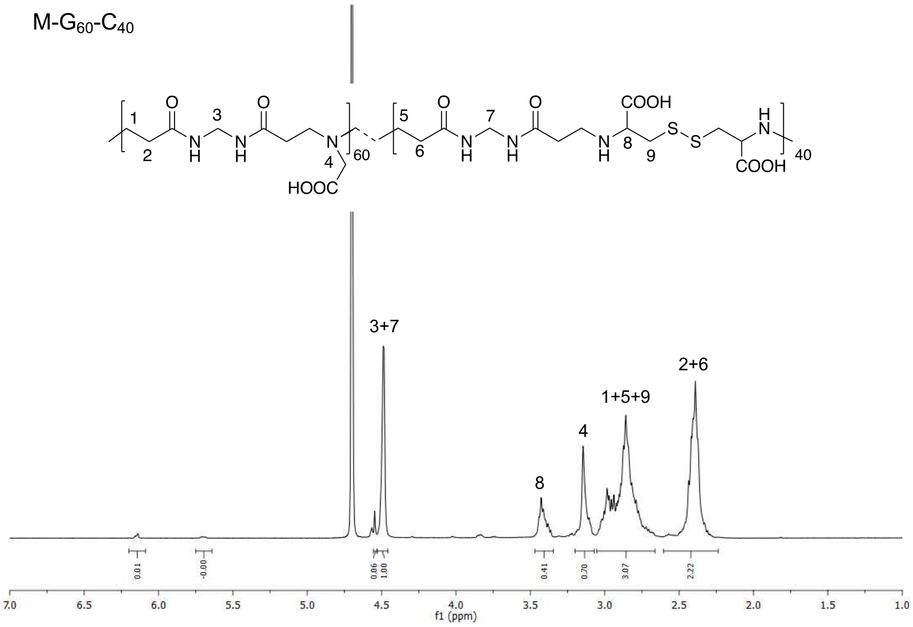

Figure S4. ¹H NMR spectrum of M-G_{60}-C_{40} copolymer.

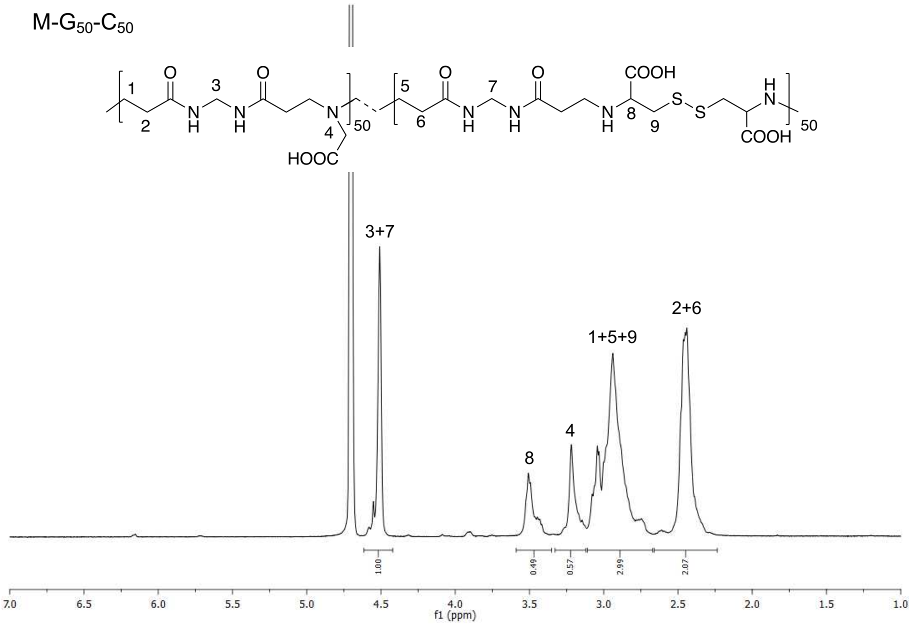

Figure S5. ^{1}H NMR spectrum of M-G_{50}-C_{50} copolymer.

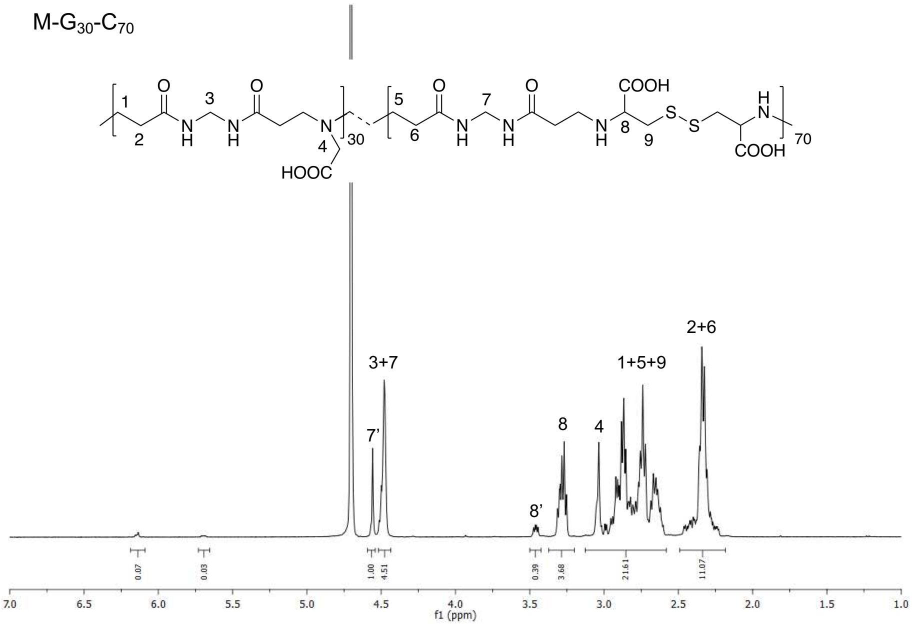

Figure S6. ^{1}H NMR spectrum of M-G_{30}-C_{70} copolymer.

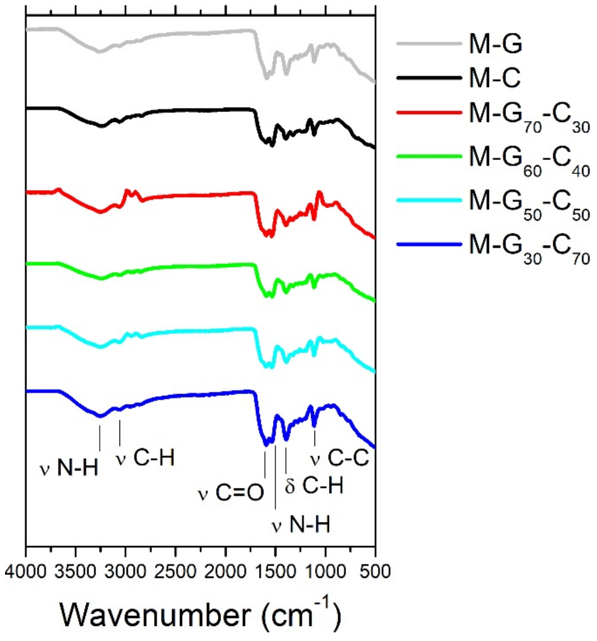

Figure S7. FT-IR/ATR of PAA homopolymers and copolymers.

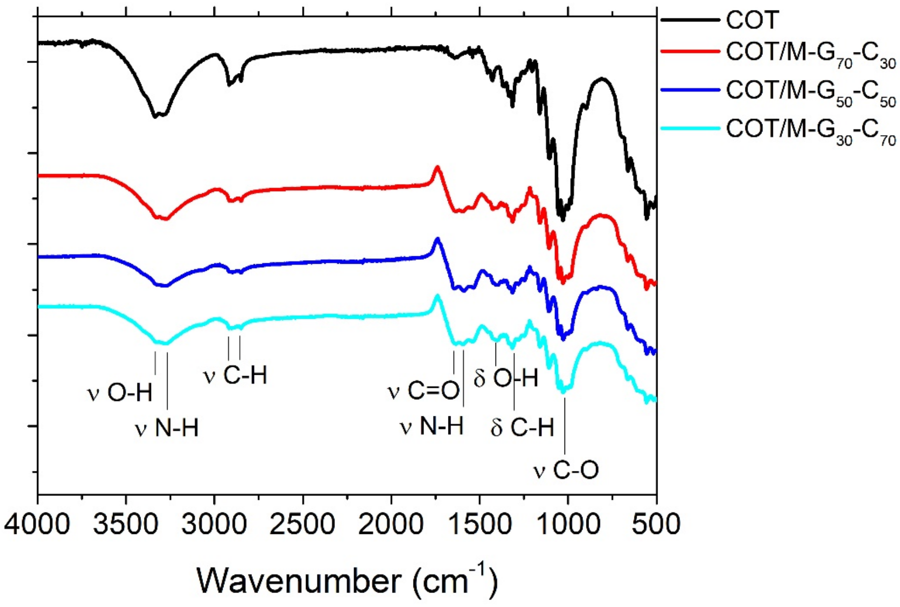

Figure S8. FT-IR/ATR of cotton untreated and treated with copolymeric PAAs.

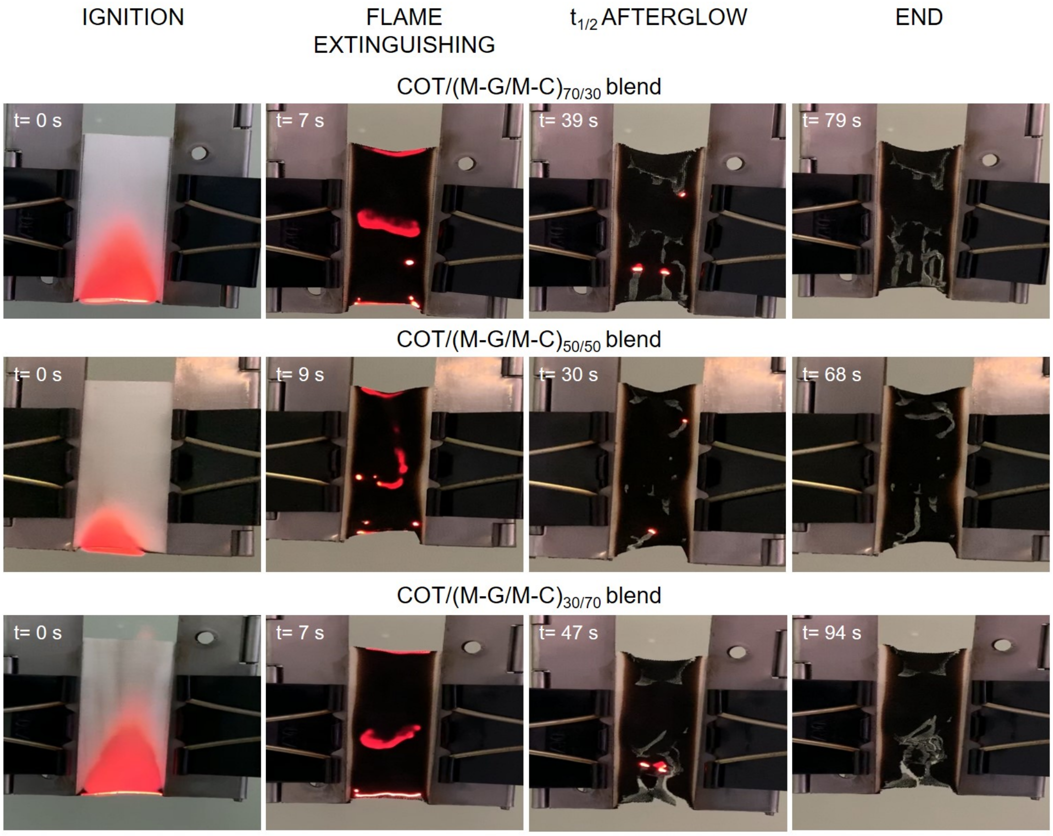

Figure S9. Snapshots of cotton fabrics treated with (M-G/M -C)$_{70/30}$ (add-on: 16.2%), (M-G/M-C)$_{50/50}$ (add-on: 16.5%) and (M-G/M-C)$_{30/70}$ (add-on: 16.5%) blends in vertical flame spread tests.
